# Can metabolomics in addition to genomics add to prognostic and predictive information in breast cancer?

**DOI:** 10.1186/1741-7015-8-73

**Published:** 2010-11-16

**Authors:** Anthony Howell

**Affiliations:** 1 Manchester Breakthrough Breast Cancer Research Unit, School of Cancer and Enabling Studies, University of Manchester, Paterson Institute for Cancer Research, Wilmslow Road, Manchester M20 4BX, UK

## Abstract

Genomic data from breast cancers provide additional prognostic and predictive information that is beginning to be used for patient management. The question arises whether additional information derived from other 'omic' approaches such as metabolomics can provide additional information. In an article published this month in *BMC Cancer*, Borgan *et al. *add metabolomic information to genomic measures in breast tumours and demonstrate, for the first time, that it may be possible to further define subgroups of patients which could be of value clinically.

See research article: http://www.biomedcentral.com/1471-2407/10/628

## Introduction

The major problems in breast cancer are predicting women at risk more precisely and predicting the presence of micrometastases at the time of primary surgery, including whether they will grow in the future and their responsiveness to systemic therapy. Transcriptomics have led to improvements of standard prognostic markers such as tumour size and axillary lymph node status. In parallel, kits for delineating expression of selected predictive and prognostic gene expression profiles have been developed and are commercially available. Clinical trials (TAYLORX and MINDACT) are in progress to determine their value for selection of appropriate adjuvant systemic hormone and chemotherapy. However, the question arises whether other 'omics' such as proteomics and metabolomics can add to the prognostic and predictive information already available from genomics given the heterogeneity and remaining behavioural unpredictability of breast tumours, as well as whether such studies might indicate additional therapeutic targets or whether adding 'omic' platforms together may be clinically useful? The paper by Borgan *et al*. [1], published this month in *BMC Cancer*, from two centres in Norway is the first attempt to assess the interactive value of transcriptomics and metabolomics in a series of primary breast cancers.

## Metabolomics

Metabolomics is the study of the metabolic changes which occur in living systems as a result of gene and protein expression and may enhance the information provided by genomics and proteomics. The metabolome may be the most sensitive measure of cellular phenotype, and methods are evolving to measure the metabolome of a single cell. Analytical methods for metabolomics analysis include liquid chromatography-mass spectroscopy (hundreds of metabolites with multiple unknown peaks), gas chromatography-mass spectroscopy (GC-MS approximately 120 to 200 metabolites) and the method used in the paper by Borgan *et al. *which uses high resolution magic angle spinning, magnetic resonance spectroscopy (HR-MAS MRS. approximately 20 to 40 metabolites) [2]. The advantage of the latter technique is that it can be carried out on standard preparations of tissues without tissue extraction and the derivatisation necessary for GC-MS. In addition, the results can be available in less than one hour, although the assays have less resolution and sensitivity compared with the more time-consuming GC-MS.

## Metabolomics in cancer

Previous studies have demonstrated that metabolomic analysis can distinguish between cancer and non-cancer tissues but do not readily distinguish grade and stage [3-7]. In the current study, Borgan *et al. *focussed mainly on oestrogen receptor positive (ER + ve) tumours defined as Luminal A type by genomic analysis. ER + ve breast cancers are the largest group of invasive disease and there is a need to distinguish those that will and will not respond to hormone therapy for a plethora of reasons. Metabolomic analysis indicated that the Luminal A subtype could be separated into three groups using multivariate analysis and hierarchical clustering. The metabolites which helped distinguish between the three groups included α and β glucose aminoacids, myo-inositol and lipid residues. Gene ontology (GO) enrichment analyses using Gene Set Analysis (GSA) indicated that one subtype of luminal A was enriched for biological processes related to cell cycle and DNA repair and thus may be a group resistant to hormone therapy. The investigators also assessed the levels of eight metabolites (high to low) in relation to the transcriptional activity of each ER + ve tumour. Myo-inositol and taurine high, ranked with GO terms related to extracellular matrix and choline high was associated with GO terms related to the cell cycle such as 'cell cycle process' and 'chromosome segregation'. The results summarised above and discussed in more detail by Borgan and colleagues this month in *BMC Cancer *[1] are novel because of the reported analytical interactions between genomic and metabolomic results. Their clinical significance will be shown when analyses are performed on frozen tumours saved from patients with long-term follow up.

One general analytical problem with this approach is the variable amount of epithelium and stroma in the samples, since recent evidence suggests marked differences and interactions between the metabolism of tumour cells and tumour associated fibroblasts [7]. In addition, it may also be difficult to make definitive statements concerning the value of the metabolome without also assessing additional metabolites using more sensitive techniques such as GC-MS [4]. Also, since we know from genomic studies that the stroma gives prognostic and predictive information over and above that given by the epithelium, there is a need to explore measuring metabolites after separation of these two compartments in tumours. Since only relatively small tumour samples are required it should be possible to assess the effects of various treatments on the metabolome in the interval between biopsy and definitive surgery two to three weeks later: so-called, 'window studies'.

## Conclusions

Using metabolomics to improve prognostic prediction in cancer looks to be a promising approach, however, further studies are required to determine the precise value of metabolomics in the management of patients with breast cancer.

## Abbreviations

HR-MAS MRS: high resolution magic angle spinning, magnetic resonance spectroscopy; GC-MS: gas chromatography-mass spectroscopy; ER + ve: oestrogen receptor positive.

## Competing interests

The author declares that they have no competing interests.

## Pre-publication history

The pre-publication history for this paper can be accessed here:

http://www.biomedcentral.com/1741-7015/8/73/prepub
